# Acquiring musical knowledge increases music liking: Evidence from a neurophysiological study

**DOI:** 10.1002/pchj.791

**Published:** 2024-07-21

**Authors:** Yingying Hou, Bei Song, Yi Zhu, Linwei Yu, Yi Hu

**Affiliations:** ^1^ Shanghai Key Laboratory of Mental Health and Psychological Crisis Intervention, School of Psychology and Cognitive Science East China Normal University Shanghai China; ^2^ Department of Art Harbin Conservatory of Music Harbin China

**Keywords:** knowledge similarity, music liking, musical knowledge, physiological similarity, skin conductance

## Abstract

People possessing musical knowledge tend to enjoy music more, but the linkage remains to be determined. Based on the shared affective motion experience model for music appreciation, we hypothesized that acquiring musical knowledge about the music itself, for example, an analytical understanding of music elements and the related emotional expressions, would increase music liking. To test the hypothesis, we asked 48 participants to learn analytical or historical information about a piece of music by watching a pre‐recorded teaching video. Learners' physiological responses, such as skin conductance and heart rate, were recorded during learning. The increase of music liking was observed after both types of knowledge acquisition, but more so for analytical knowledge. Notably, acquiring analytical knowledge made learners' skin conductance more similar, indicating the alignment of physiological responses. This physiological similarity, correlated with analytical knowledge similarity, could mediate the effect of knowledge acquisition on music liking. In sum, this study reveals the impact of analytical knowledge on music enjoyment and the associated neurophysiological mechanism. It extends the theoretical framework of shared affective motion experience to explain how musical knowledge influences music appreciation.

## INTRODUCTION

Music is an integral part of our daily life, on par with primary rewards like food (Martinez‐Molina et al., 2016). It can evoke emotions such as joy, fear, sadness, and memories of personal experiences. For example, listening to music at a graduation ceremony excites us and brings back some vivid past memories. However, existing literature has found mixed results on whether previously acquired knowledge influences music liking (Halpern, 1992; Larson, 1971; Price & Swanson, 1990; Williamson‐Urbis, 1995; Woody & Burns, 2001). On the one hand, having more musical knowledge, such as those about musical styles, can increase music liking (Madison & Schiölde, 2017). On the other hand, musical knowledge can also reduce novelty, or interfere with emotional responses to music, and decrease music liking (Ackermann & Merrill, 2022). Therefore, the relationship between musical knowledge and enjoyment is unclear. More research is needed to examine whether and how musical knowledge is related to music liking.

There are different types of knowledge. One type is about music pieces, such as knowledge of analyzing music elements, while another is about general music contexts, such as knowledge of musical history (Halpern, 1992). Analytical knowledge comprises information such as pitch, rhythm, and melody, and methods of evaluating the quality and beauty of musical performance. It helps us understand how music conveys its meaning (Halpern, 1992). For example, using the concepts of pitch class set, we can know how music elements are related in non‐tonal music (Chapman, 1978). Using Schenkerian analysis, we know the underlying structure and voice leading of tonal music, such as Bach's works (Marsden, 2010). Analytical knowledge can also help us appreciate the expressiveness of music pieces compared with other pieces.

In contrast, historical knowledge includes information about the origin and development of musical styles, genres, performances, and so forth. It helps us understand how music relates to the social environment and its impact (Halpern, 1992). For example, by learning the historical context of Beethoven's symphonies, we can appreciate how they expressed the ideals of heroism in the turbulent times of the French Revolution (Lockwood, 2015). By studying the historical background of jazz music, we understand how it inspired artistic innovations in the twentieth century (Gioia, 2021). Historical knowledge can also help us discover the story behind a piece of music, such as the composer's motivation, inspiration, and the audience's evaluation.

Analytical knowledge may substantially influence emotion and preference for music pieces compared with historical knowledge. According to the shared affective motion experience (SAME) model (Molnar‐Szakacs et al., 2009; Overy & Molnar‐Szakacs, 2009), music appreciation involves perceiving the emotional expressions conveyed by performers through acoustic cues (e.g., tempo, loudness). Performers use acoustic cues, such as tempo, loudness, and timbre, to convey emotions. These cues can affect how listeners perceive the music and evaluate the performance. For example, faster tempo, higher loudness, and brighter timbre are often associated with positive emotions, for example, happiness. In comparison, slower tempo, lower loudness, and darker timbre are related to negative emotions, for example, sadness (Juslin & Laukka, 2004). Some music performers are exceptionally skilled at using these cues to convey emotions to audiences (Juslin, 2000). For example, Luciano Pavarotti was praised for using loudness to express passion in his operatic singing. Listeners detect these cues and experience the conveyed emotions (Gabrielsson & Juslin, 1996; Juslin & Laukka, 2003). Active emotional engagement is related to greater music enjoyment (Diaz, 2015; Juslin, 2013a, 2013b; Zalanowski, 1986). In essence, the SAME model emphasizes the importance of perceiving musical sounds and emotional expressions (Madsen, 1999; Reimer, 2003). Based on this model, analytical knowledge that focuses on the music and its emotional expressions may elicit more music enjoyment. Moreover, such knowledge about how musical elements create emotional meaning can help listeners better detect expressed emotions. This understanding fosters shared cognition and affective attunement among listeners, thereby enhancing their music experience.

Listening to music influences the listener's physiological reactions. It can change heart rate and skin conductance, which are indicators of arousal and emotion (Blood & Zatorre, 2001; Juslin & Sloboda, 2011; Mas‐Herrero et al., 2014; Salimpoor et al., 2009; Terry et al., 2020). For instance, listening to fast‐tempo, high‐volume music (such as certain types of rock music) can accelerate heart rate and make listeners feel more aroused, whereas listening to slow‐tempo, low‐volume music (such as certain types of classical music) may decelerate heart rate and make listeners feel more relaxed (Lingham & Theorell, 2009; Schafer et al., 2013; Smith & Joyce, 2004). Some research has found that listeners have concordant changes in skin conductance when watching live music performances; physiological concordance is related to similar emotional experiences and enjoyment of music (Chabin et al., 2022; Czepiel et al., 2021; Tschacher et al., 2023). Likewise, physiological concordance occurs during the acquisition of knowledge. Learners in the classroom demonstrated synchronous fluctuations in skin conductance, relating to their emotional state and level of engagement (Gashi et al., 2018; Zhang et al., 2021). The occurrence of physiological concordance both in music listening and knowledge acquisition suggests a link between them.

In the current study, we aimed to determine the effects of different musical knowledge on music enjoyment. Existing literature has revealed an important role of “context” or “source” information in influencing music appreciation (Thompson et al., 2023). For instance, by manipulating details about the performer, program notes, titles or origins of the music pieces, previous studies have shown that such contextual cues significantly affect listeners' evaluation of the same music performance (e.g., Anglada‐Tort & Müllensiefen, 2017; Anglada‐Tort et al., 2021; Aydogan et al., 2018; Kroger & Margulis, 2017). While these studies demonstrate the effect of contextual information on music judgment in general, no study so far has systematically examined the distinction between analytical (i.e., about core music elements and emotional expressions) versus historical (i.e., about stylistic contexts and backgrounds) types of musical knowledge. Our study aimed to address this gap by investigating how learning analytical versus historical information about the same music piece influences music appreciation.

In this study, we only included female participants. This decision was based on prior research indicating potential gender differences in music responses (Hou et al., 2020; Pan et al., 2023; Zhu et al., 2022). By focusing solely on female participants, we aimed to control for these differences and focus on the impact of knowledge type on music appreciation.

We employed a video‐based teaching paradigm, enabling participants to learn either analytical or historical knowledge. Based on the SAME model, we hypothesized that analytical knowledge, which elucidates emotional expressions within music elements, would increase music liking (Botvinick et al., 2005; Budell et al., 2010; Diaz, 2015; Zalanowski, 1986). This hypothesis stems from the premise that analytical knowledge fosters a deeper emotional understanding of music, aligning the individual emotional responses of listeners. To test this, we measured physiological responses across multiple channels to assess the degree of similarity among participants during the learning process. This could help us understand how knowledge acquisition creates embodied meaning that enhances music enjoyment. The measure of physiological similarity serves as an indicator of shared emotional experiences, a concept central to the SAME model. We anticipated that the in‐depth exploration of music's emotional aspects through analytical knowledge would lead to greater physiological concordance among learners, reflecting a collective emotional engagement with the music. Furthermore, drawing on the evidence that people watching the same emotionally expressive content exhibited similar physiological responses (Bracken et al., 2014; Golland et al., 2014; Muszynski et al., 2016, 2018), our study extends this concept to the domain of music education. We propose that the type of knowledge imparted—analytical or historical—may influence the degree of emotional synchronization in a group setting. We explore the relationships between knowledge type, physiological similarity, and music liking. Through this investigation, we aim to uncover the potential physiological mechanisms underlying how different types of musical knowledge influence music appreciation, thus providing insights into the interplay between cognitive understanding and emotional response in music enjoyment.

The above research hypotheses and experimental design address several practical questions in music education. On the one hand, two types of knowledge instructions may induce different learning changes at behavioral and physiological levels so that music educators could customize teaching strategies based on the potential learning effects. Further, our expectancy that analytical knowledge enhances music appreciation more than historical knowledge can provide practical guidance for music teachers, namely teaching more musical elements and emotional expression in classrooms. On the other hand, the video teaching examples used in this study can serve as references for music educators to design practical techniques for music knowledge instruction, such as cultivating students' essential understanding and appreciation of music. The physiological response methods used here could also be applied to evaluate the effects of music instruction and provide scientific approaches for assessing music education.

## METHOD

### Participants

Forty‐eight female participants (age: 21.48 ± 1.88 years; M ± SD) with no formal music training took part in this study. They were randomly assigned to the group of analytical knowledge (24 learners, age: 21.38 ± 2.02 years) or historical knowledge (24 learners, age: 21.58 ± 1.77 years). The two groups did not differ in age (*t*(46) = −0.38, *p* = .711). All participants were right‐handed and had no history of neurological or psychiatric disorders. Written informed consent was obtained after the experimenter explained the study. This study was approved by the University Committee of Human Research Protection (HR 147‐2019) at East China Normal University.

Given that the main interest of this study is the effect of two types of musical knowledge (analytical vs. historical), the target effect for prior power analysis should be the group differences in terms of music liking increment (from pre‐learning to post‐learning) or physiological similarity, tested by two‐sample *t*‐tests. We calculated the target sample size based on the effect size estimated during the study, with the consideration of potential upward bias (Anderson et al., 2017). Specifically, we first recruited 12 participants per group, which showed a significant group difference in music liking increment (*t*(22) = 2.70, *p* = .013, Cohen's *d* = 1.10) and physiological similarity (*t*(22) = 4.27, *p* < .001, Cohen's *d* = 1.74) between the analytical and historical knowledge groups. To account for potential upward bias in the effect sizes, we then performed a prior power analysis using the Bias and Uncertainty Corrected Sample Size (BUCSS) method (Anderson et al., 2017). With parameters of 95% power, alpha = 0.05, assurance = 0.80, and bias correction, the analysis indicated a required sample size of 18 per group to detect the estimated group difference in physiological similarity. Based on this power analysis, we determined the final sample size of 24 participants per group, which provided sufficient power to detect the target effects in this study.

### Materials

A music professor (female, 40 years of age), with over 15 years of teaching experience at the Harbin Conservatory of Music in China, selected Robert Schumann's “Träumerei” from Kinderszenen opus 15 for the study. This piece was chosen for being a world‐class classic, short in length, and unfamiliar to non‐professionals, as confirmed by a preliminary 7‐point Likert scale survey conducted on 16 other participants without musical training, revealing low familiarity (1.88) and moderate liking (3.63). It is commonly performed as a solo piano piece, but has also been adapted for other instruments such as violin, cello, and harmonica. The choice of a single music piece aimed to avoid knowledge interference between pieces, in line with findings from prior research on the negative impact of extraneous information on learning (Kaminski & Sloutsky, 2013; Paas et al., 2003).

Along with selecting the piece, the professor provided two 10‐min lessons on analytical and historical knowledge, video‐recorded for 689 and 627 s, respectively. The analytical teaching unpacks the music itself—its emotional theme, instrument timbre, tempo/dynamics features, and form structure. Excerpts of the appreciated music piece are inserted during teaching. The historical knowledge teaching focuses on relevant musical works, composers, and backgrounds that help appreciate the artistic style and value of the music piece (see details in Supplementary Materials).

To validate the comparability of the analytical and historical teaching contents, we evaluated the text transcripts of the teachings. Another group of 13 participants read the two text transcripts in a counterbalanced order, rating each on interest, appeal, and emotional arousal and valence using a 7‐point Likert scale. Statistical comparisons revealed no significant differences between knowledge conditions on these ratings (Wilcoxon *z*s <1.59, *p*s > .112), indicating comparable interest, appeal, and emotions between the two teaching contents.

A skilled violinist (male, 23 years of age) was invited to perform the selected music piece and his performance was recorded by a video machine (190 s; see details in Supplementary Materials). He had undergone 18 years of rigorous violin training and obtained the highest artistic level certificate (Level 12) in violin performance from the prestigious Shanghai Conservatory of Music in China.

### Experimental protocol

**FIGURE 1:**
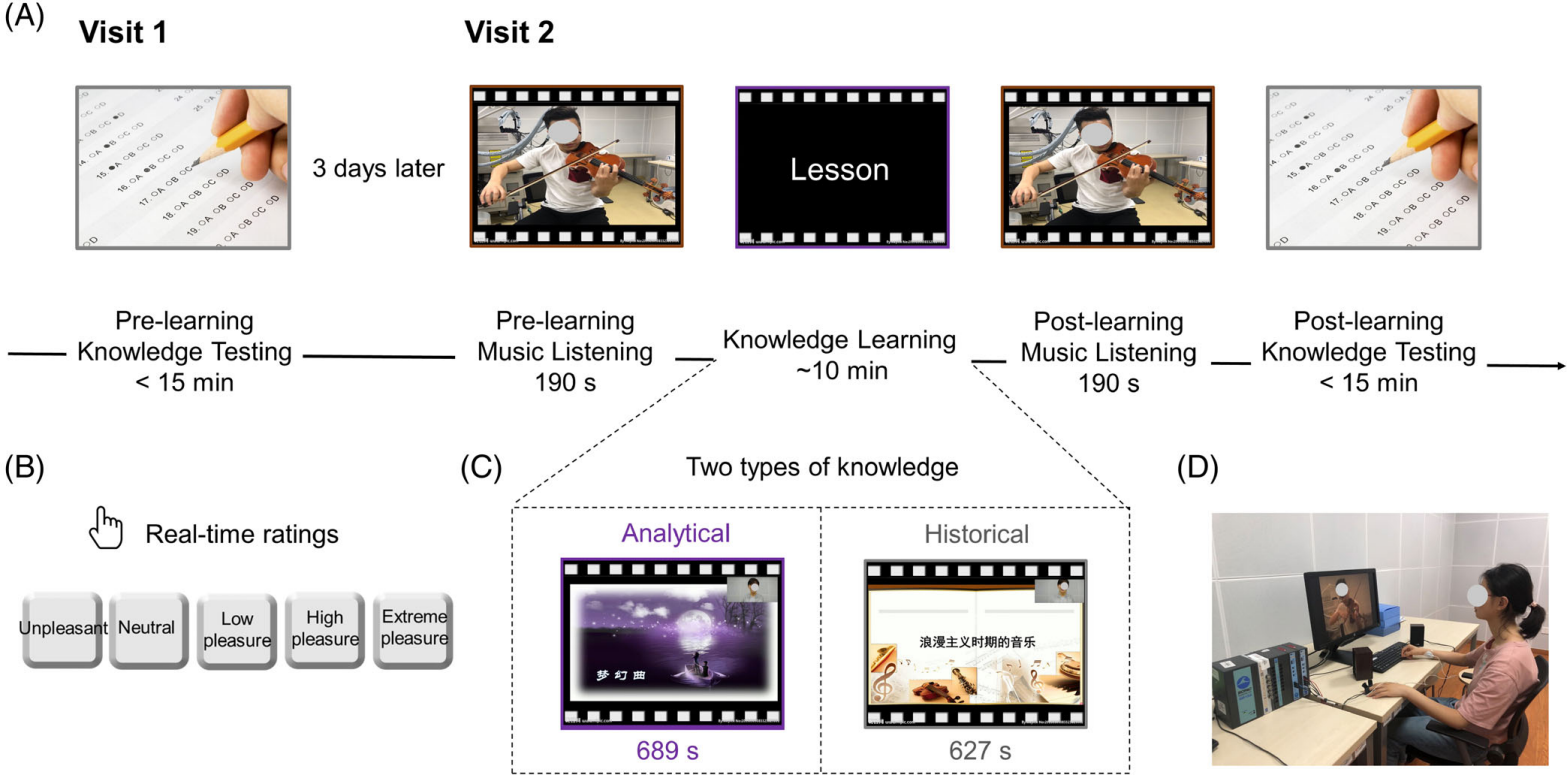
Experimental protocol. (A) In Visit 1, participants completed pre‐learning knowledge tests. In Visit 2, participants completed three experimental tasks of pre‐learning music listening, information learning, and post‐learning music listening. (B) During the music listening tasks, participants listened to a pre‐recorded video of a violin performance and were instructed to report their pleasure level in real time. (C) In the information learning task, participants viewed a pre‐recorded teaching video on either analytical or historical knowledge. (D) Each participant's physiological activities of skin conductance and heart rate were recorded during the experimental tasks.

Each participant visited our lab two times at a 3‐day interval (see Figure 1A).

During Visit 1, participants completed two tests (< 15 min) to evaluate their prior analytical and historical knowledge. Each test comprised five single‐choice questions and five true‐or‐false questions. The tests were collaboratively designed by the professor and the experimenter. Moreover, nine additional participants were recruited to assess the test difficulty of the analytical and historical knowledge (scores: 3.67 ± 1.73 vs. 4.56 ± 1.59, Wilcoxon *z* = −1.35, *p* = .176). An online version of the Jake Mandell Tone Threshold Test (JMTT) (Mandell et al., 2007) was implemented to evaluate participants' musical ability (http://jakemandell.com/tonedeaf/). This brief test produces scores ranging from 0 to 100%, with higher percentages indicating better pitch discrimination and musical memory. While it is not as widely used as other standardized music perception assessments, statistical analysis of over 60,000 past test takers supports the validity of the JMTT for indexing musical pitch discrimination skills. The challenging test items help avoid the clustering of high scores solely among individuals with exceptional musical abilities. In addition, a 5‐point Likert scale, known as the Interpersonal Reactivity Index (IRI), assessed the participants' cognitive and emotional empathy abilities (Davis, 1980). There were no significant differences in musical or empathy abilities between groups (*t*s <1.21, *p*s > .231).

During Visit 2, participants were guided into a quiet room. The experimenter gave detailed instructions about the upcoming experimental tasks. Participants' physiological activities, including skin conductance and heart rate, were monitored using a multichannel electrophysiological recording device attached to their fingers, wrist, and ankles. They were given 3 min for an initial rest, during which they were asked to relax with their eyes open. Next, they undertook three tasks, that is, pre‐learning music listening, knowledge learning, and post‐learning music listening.

In the pre‐learning music listening task, participants watched and listened to the pre‐recorded video described above. They were instructed to focus on the violinist's face and press one of five keyboard buttons to report their pleasure level (1 = *unpleasant*, 2 = *neutral*, 3 = *low pleasure*, 4 = *high pleasure*, 5 = *extreme pleasure*; see Figure 1B). They were told to press the button down with their right hand at the onset of pleasure and hold it until their feelings changed. During music listening, the experience of pleasure might occur several times or might not occur at all. Whenever feeling pleasure, they indicated it by pressing the corresponding button (Colver & El‐Alayli, 2016). This real‐time pleasure rating procedure has been extensively used in previous studies on music liking (Colver & El‐Alayli, 2016; Martinez‐Molina et al., 2016; Panksepp, 1995; Salimpoor et al., 2009). Note that the real‐time pleasure ratings were used to generate an index of music liking. After the listening, additional ratings were taken immediately using Likert scales: general pleasantness (from 0 = *none* to 10 = *intense*), emotional arousal (from 0 = *very relaxing* to 10 = *very arousing*), valence (from 0 = *very sad* to 10 = *very happy*), and empathy for the entire music piece (5 items; see details in Supplementary Materials). These ratings were completed via a paper questionnaire. A 2‐min rest followed the ratings.

Next, participants conducted the knowledge acquisition task by watching the pre‐recorded video of teaching analytical or historical knowledge described above (see Figure 1C). They were instructed to listen carefully. After watching, they rated emotional arousal and valence, learning difficulty and engagement, and teacher likability (from 0 = *not very much* to 10 = *very much*). Following this, they completed the same knowledge tests as in Visit 1. A 2‐min rest followed the tests.

Finally, in the post‐learning music listening task, participants listened again to the same piece of music as in the pre‐learning task and re‐rated their real‐time pleasure. They also complete the same additional ratings as before, that is, general pleasantness, emotional arousal, valence, and empathy for the music.

### Physiological recording and preprocessing

The experiment continuously monitored participants' physiological responses using Biopac MP150 (Biopac Systems Inc., CA, USA; see Figure 1D). Skin conductance data were collected by attaching electrodermal electrodes to the left index and middle fingers. Heart rate data were acquired using electrocardiogram electrodes placed on the right ankle, left ankle, and left wrist. Data were collected at a sampling rate of 250 Hz using Acknowledge software 5.0. Afterward, each participant's continuous physiological data during knowledge acquisition were extracted for analysis. Notably, data during music listening were not reported here.

Raw skin conductance data were filtered using median smoothing with 1‐s width and a low‐pass filter set at 1 Hz. Due to sensor errors causing data loss, one participant in the analytical knowledge group was excluded from the skin conductance analysis, leaving 23 participants.

Heart rate data were analyzed using the freely available ARTiiFACT 2.0 software (Kaufmann et al., 2011) (Psychonomic Society, Inc.) to detect inter‐beat intervals (IBIs). We visually accessed the success of beat position detection and excluded two participants in the historical knowledge group due to poor data quality. We then used an algorithm to detect and correct artifacts via cubic spline interpolation (Berntson et al., 1990). These preprocessed IBI data were transformed into a time series by interpolating consecutive intervals and resampling at 32 Hz.

## DATA ANALYSIS

### Analysis of knowledge acquisition

Both groups of participants completed two tests of analytical and historical knowledge. We calculated pre‐ and post‐learning knowledge test scores for each test based on 10 questions answered correctly. For each group, a two‐way repeated measures analysis of variance (ANOVA) was then conducted on the knowledge scores with the within‐participant factors of Time (pre‐learning vs. post‐learning) and Knowledge (analytical vs. historical). Post‐hoc multiple testing was performed with Bonferroni‐corrected *p*‐values.

To determine whether non‐significant results indicate evidence for null effects or are indeterminate, we compared the evidence for null (H0, e.g., knowledge score did not increase post‐learning) versus the alternative (H1, e.g., knowledge score did increase post‐learning) hypotheses by using the Bayes factor (BF). BF_10_ is computed to quantify evidence for H1 versus H0. A BF_10_ value greater than 3 indicates substantial evidence for H1, and a value less than 1/3 indicates substantial evidence for H0 (Jeffreys, 1961). For all Bayesian tests, we used default priors provided by JASP v.0.17.0.0, which is recommended when prior knowledge is not specific or difficult to elicit (Verschuere et al., 2023). For *t*‐tests, the default prior is defined by a Cauchy distribution centered on a zero effect size (δ) and a width of 0.707. For ANOVAs, the width (of 0.50) is set so that it mimics the default prior of the *t*‐test.

### Analysis of music liking

Previous research has shown that persistent pleasurable responses during music listening indicate greater enjoyment (Colver & El‐Alayli, 2016; Panksepp, 1995; Salimpoor et al., 2009). We recorded real‐time pleasure ratings throughout listening. We analyzed occurrences of the most pleasure responses (ratings 4 and 5 for high and extreme pleasure, respectively) and quantified music liking as the number of these responses multiplied by weighted values (rating 4 = 1; rating 5 = 2).

We aimed to determine if knowledge acquisition increased music liking. A two‐way mixed‐design ANOVA was conducted on music liking, with Group (analytical vs. historical knowledge learning group) as a between‐participant factor and Time (pre‐learning vs. post‐learning) as a within‐participant factor. Post‐hoc multiple comparisons were adjusted with the Bonferroni correction and Bayesian tests were used. Importantly, comparing pre‐ versus post‐music liking could mitigate the influence of individual response tendencies, such as those tending to make frequent high or low responses.

### Physiological data analysis

Physiological similarity was used to estimate the relationship between each participant's physiological activities and others in the same group. We computed physiological similarity using the method described by the study by Marci et al. (2007). The preprocessed physiological data were analyzed in the following steps.

First, for each participant, we computed the average slope of data within a moving 5‐s window. The window was then moved forward by 1 s, and the next 5‐s slope value was calculated, resulting in successive 5‐s slope values in 1‐s increments. Second, for each pair of participants, we calculated the zero‐lag Pearson correlations over consecutive, running 15‐s windows (corresponding to 15 slope averages). Third, a global index of physiological similarity for each pair was computed by estimating the ratio of the sum of positive correlations divided by the sum of the absolute value of negative correlations. To account for skew related to ratios, we applied a natural logarithmic transformation to each pair's indices (Chabin et al., 2022). Finally, the similarity values between each participant and all other participants in the same group were averaged. One‐sample *t*‐tests were performed on the averaged physiological similarity. A value greater than zero indicates more similar physiological responses between participants over time. Two‐sample *t*‐tests were used to compare physiological similarities between knowledge groups.

We conducted a permutation test to discern non‐task‐specific physiological similarities that could arise, for instance, from commonalities in the physical environment during a particular experimental session or from general increases in participants' arousal. This disrupted the fine‐grained temporal correspondence between participants' physiological signals and included the following steps. First, each participant's physiological data were segmented into 1‐s epochs. We then randomly shuffled these epochs over time, creating a set of temporal‐shuffled epochs. Using the permuted datasets, physiological similarity values across participants were calculated. A one‐sample *t*‐statistic was obtained based on these similarity values. Finally, the above steps were repeated 1000 times (i.e., 1000 permutations) to generate a null *t* distribution. We compared the observed *t‐*value to this distribution and calculated the statistical significance (i.e., *p*‐value) using the formula erfc((|*s* − *μ*_0_|/*σ*_0_)/√2) (Theiler et al., 1992), where *s* denotes the observed *t‐*statistic, and *μ*_0_ and *σ*_0_ represent the mean and standard deviation of the null distribution, respectively.

### Analyses on relation between physiological similarity and music liking change

We then explored the possible relations between physiological similarity and increased music liking using correlation and mediation analyses. We calculated music liking change by subtracting pre‐ from post‐learning scores. Pearson correlation analysis was used to assess the association between physiological similarity and music liking change. To determine if this association was independent of the type of knowledge learned, we conducted multiple regression analysis with *Group* as a control variable.

The mediation analysis was performed using SPSS 25.0 and SPSS PROCESS macro 4.1 software (Hayes, 2013). We constructed the mediation model based on the following regression equations (MacKinnon et al., 2007):

(1) $$Y=\mathrm{cX}+e_{y}$$

(2) $$M=\mathrm{aX}+e_{m}$$

(3) $$Y=c^{'}X+\mathrm{bM}+e{'}_{y}$$

where *X* represents the independent variable (type of information learned, dummy coded 0 for historical knowledge and 1 for analytical knowledge), *M* is the mediator (physiological similarity), *Y* is the dependent variable (music liking change), and *e* denotes residuals. Path *a* represents the regression coefficient relating *X* to *M*, while path *b* represents the regression coefficient relating *M* to *Y*, adjusted for *X*. Paths *c*′ and c describe the relationship of *Y* and *X* with and without *M*, respectively. We conducted the mediation analysis with 5000 bootstrapping repetitions. The results were considered significant if the 95% confidence interval (CI) did not include 0.

### Analysis on relation between physiological similarity and knowledge similarity

We calculated knowledge similarity between a participant and others in the same group in a similar way to the calculation of physiological similarity described above.

First, we coded each participant's responses to all 10 test questions as either 1 (correct) or 0 (incorrect), generating a 10‐dimensional vector of test performance. For each pair of participants, we calculated the cosine similarity between their two test performance vectors. Cosine similarity measures the similarity between vectors in multidimensional space and determines whether two vectors point in roughly the same direction (Han et al., 2011). It is calculated by the cosine of the angle between two vectors using the following formula:

$$\text{similarity}\left(x,y\right)=\cos\left(\theta \right)=\frac{x\cdot y}{\left\|x\right\|\left\|x\right\|}$$

here, ||*x*|| refers to the Euclidean norm of vector **x** = (*x*_1_, *x*_2_, …, *x*_*p*_), defined as *x*_12_ + *x*_22_ + … + *x*_*p*2_. Conceptually, it represents the length of the vector. Likewise, ||*y*|| is the Euclidean norm of vector **y**. This measurement computes the cosine of the angle between vectors **x** and **y**. A cosine value of 0 means that the two vectors are at 90 degrees to each other (orthogonal) and have no match. The closer the cosine value to 1, the smaller the angle and the greater the match between vectors. We averaged the cosine similarities between each participant and others in their group to obtain a knowledge similarity index for that individual. Higher scores indicated sharing more knowledge with others in the group. Finally, we used Pearson correlation analysis to assess the relationship between physiological and knowledge similarity.

### Data, materials, and software availability

The data, materials, and analytic code in this article are available at https://osf.io/yqdwk/.

## RESULTS

### Knowledge acquisition in analytical and historical groups

We first examined whether learning groups acquired the expected knowledge. Before and after learning, participants completed the test consisting of analytical and historical knowledge. A two‐way repeated measure ANOVA was conducted for each group, with the within‐subject factors of Knowledge (analytical vs. historical) and Time (pre‐learning vs. post‐learning).

**FIGURE 2:**
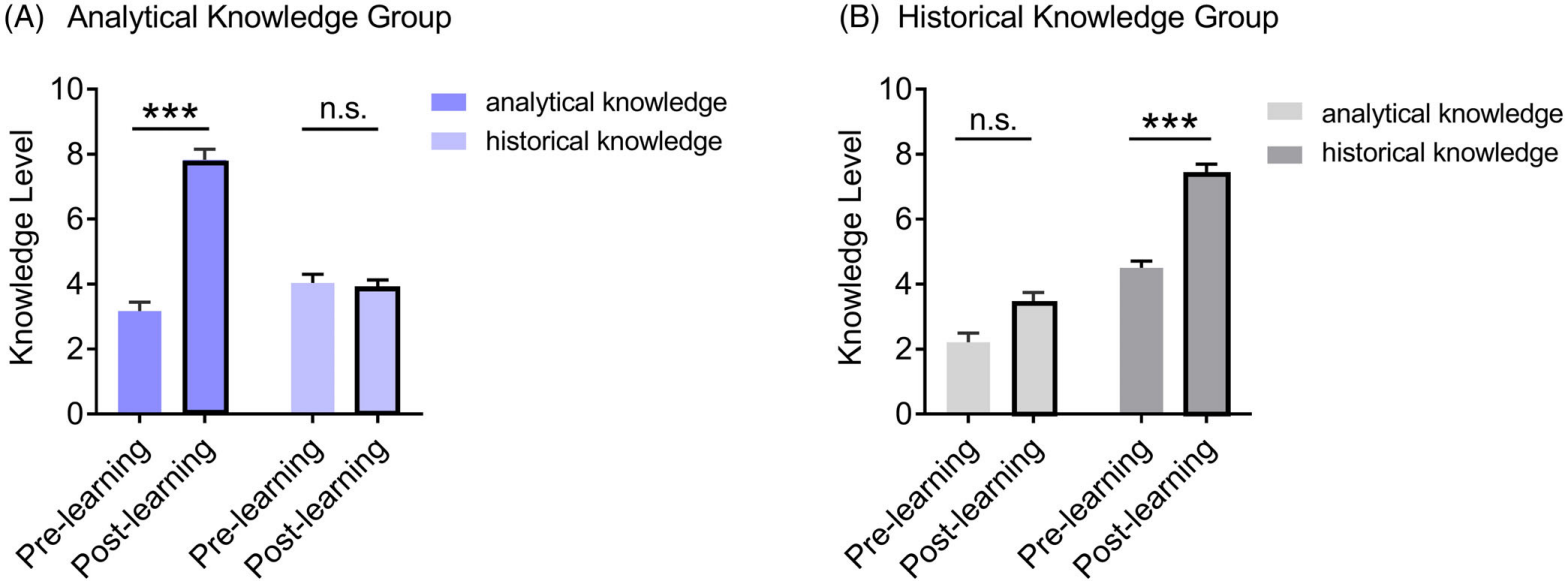
Knowledge level of two groups before and after learning. (A) Participants in the analytical knowledge learning group significantly improved their analytical knowledge level. The increase in analytical knowledge was significantly greater than that of historical knowledge. (B) Participants in the historical knowledge learning group significantly improved their historical knowledge level. The increase in historical knowledge was significantly greater than that of analytical knowledge. Error bars represent standard errors of the mean, and “n.s.” indicates non‐significant (*p* > .05). ***Bonferroni‐corrected *p* < .001.

For the analytical knowledge learning group, the results showed significant main effects of Time (*F*(1,23) = 91.62, *p* < .001, *η* ^2^_partial_ = 0.80, BF_10_ = 1.11 × 10^4^) and Knowledge (*F*(1,23) = 18.49, *p* < .001, *η* ^2^_partial_ = 0.45, BF_10_ = 34.50), and their interaction effect (*F*(1,23) = 154.74, *p* < .001, *η* ^2^_partial_ = 0.87, BF_10_ = 8.42 × 10^15^). Follow‐up analyses demonstrated that this group significantly improved performance on the analytical knowledge test (7.83 ± 1.58 vs. 3.17 ± 1.34, *t*(23) = 14, *p*_bonf_ < .001, Cohen's *d* = 2.86 [95% CI: 1.94, 3.76], BF_10_ = 7.77 × 10^9^; see Figure 2A). But there was no credible evidence supporting any improved performance on the historical knowledge test (3.88 ± 1.23 vs. 4.04 ± 1.30, *t*(23) = −0.61, *p* = .548, Cohen's *d* = −0.12 [95% CI: −0.53, 0.28], BF_10_ = 0.25). Moreover, the increase in analytical knowledge was significantly greater than that in historical knowledge (*t*(23) = 12.44, *p* < .001, Cohen's *d* = 2.54 [95% CI: 1.70, 3.36], BF_10_ = 7.93 × 10^8^), indicating that the analytical knowledge learning group significantly gained more analytical knowledge compared with historical knowledge.

For the historical knowledge learning group, the results showed significant main effects of Time (*F*(1,23) = 74.18, *p* < .001, *η* ^2^_partial_ = 0.76, BF_10_ = 9.45 × 10^4^) and Knowledge (*F*(1,23) = 174.87, *p* < .001, *η* ^2^_partial_ = 0.88, BF_10_ = 3.03 × 10^8^), as well as a significant interaction effect (*F*(1,23) = 6.69, *p* = .017, *η* ^2^_partial_ = 0.23, BF_10_ = 44.97). Follow‐up analyses revealed that this group demonstrated a significant improvement in performance on the historical knowledge test (7.42 ± 1.35 vs. 4.50 ± 1.06, *t*(23) = 7.40, *p*_bonf_ < .001, Cohen's *d* = 1.51 [95% CI: 0.91, 2.09], BF_10_ = 9.71 × 10^4^; see Figure 2B). The performance improvement on the analytical knowledge test did not reach statistical significance after multiple comparison correction (3.42 ± 1.61 vs. 2.21 ± 1.38, *t*(23) = 2.87, *p*_bonf_ = .054, Cohen's *d* = 0.59 [95% CI: 0.15, 1.01], BF_10_ = 5.45). Furthermore, participants in the historical knowledge group gained significantly more historical knowledge than analytical knowledge (*t*(23) = 2.59, *p* = .017, Cohen's *d* = 0.53 [95% CI: 0.10, 0.95], BF_10_ = 3.19).

Next, we explored whether there were other differences between the two groups. A series of two‐sample *t*‐tests were conducted on the measurements done only after learning. There was little evidence for group differences in emotional arousal, valence, learning engagement, or teacher likability after learning (*t*s <1.34, *p*s > .188, Cohen's *d*s <0.39, BF_10_s < 0.60). Participants in the historical knowledge group reported higher levels of learning difficulty compared to those in the analytical knowledge group (4.38 ± 2.32 vs. 2.63 ± 2.18, *t*(23) = 2.69, *p* = .010, Cohen's *d* = 0.78 [95% CI: 0.19, 1.36], BF_10_ = 4.91).

### Music liking change from pre‐learning to post‐learning

**FIGURE 3:**
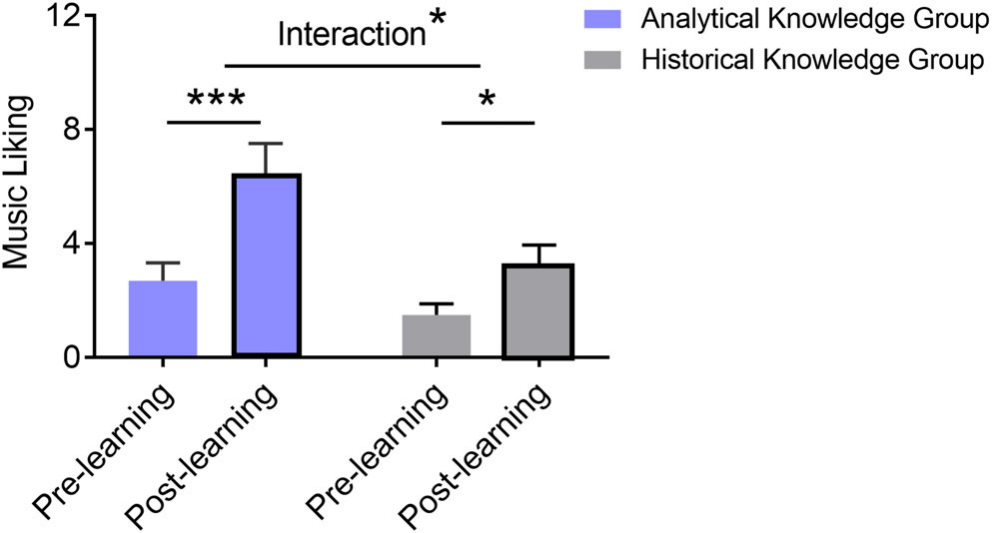
Changes in music liking for two groups before and after learning. Both groups showed a significant increase in music liking from pre‐learning to post‐learning. Notably, the increase was significantly greater in the analytical knowledge group than in the historical knowledge group. Error bars represent standard errors of the mean. *Bonferroni‐corrected *p* < .05, ***Bonferroni‐corrected *p* < .001.

We then explored the extent to which musical knowledge influences liking. A two‐way mixed‐design ANOVA was firstly conducted on music liking, with Group (learning groups of analytical vs. historical knowledge) as a between‐subject factor and Time (pre‐learning vs. post‐learning) as a within‐subject factor. The results showed significant main effects of Time (*F*(1,46) = 41.91, *p* < .001, *η* ^2^_partial_ = 0.48, BF_10_ = 8.47 × 10^4^; see Figure 3) and Group (*F*(1,46) = 5.62, *p* = .022, *η* ^2^_partial_ = 0.11, BF_10_ = 2.94), as well as a significant interaction effect (*F*(1,46) = 5.23, *p* = .027, *η* ^2^_partial_ = 0.10, BF_10_ = 2.01; see Figure 3). Follow‐up analyses revealed that music liking increased significantly from pre‐learning to post‐learning in both groups of analytical knowledge (*t*(23) = 5.55, *p*_bonf_ < .001, Cohen's *d* = 1.13 [95% CI: 0.61, 1.64], BF_10_ = 1810) and historical knowledge (*t*(23) = 3.42, *p*_bonf_ = .012, Cohen's *d* = 0.70 [95% CI: 0.24, 1.14], BF_10_ = 16.53). A two‐sample *t*‐test further showed that the increase of music liking was significantly greater in the analytical knowledge group than in the historical knowledge group (3.75 ± 3.31 vs. 1.79 ± 2.57, *t*(46) = 2.29, *p* = .027, Cohen's *d* = 0.66 [95% CI: 0.08, 1.24], BF_10_ = 2.29).

Next, we explored how other measurements were changed before and after learning. A series of two‐way mixed‐design ANOVAs were performed on the assessments of general pleasantness and empathy towards the music piece separately. The results showed significant main effects of Time and Group (*F*s > 4.19, *p*s < .046, *η* ^2^_partial_s > 0.08, BF_10_s > 1.73). Little evidence was shown for the interaction effect of Time × Group (*F*s <3.30, *p*s > .076, *η* ^2^_partial_s <0.07, BF_10_s < 0.97), indicating that both groups reported a changed general pleasantness and empathy towards the music from pre‐learning to post‐learning.

Similar analyses were conducted on two measurements of emotional arousal and valence, tested both before and after learning. There was a significant main effect of Time (*F*s > 4.59, *p*s < .037, *η* ^2^_partial_s > 0.09, BF_10_s > 1.63). Little evidence was shown for the effect of Group (*F*s <0.95, *p*s > .334, *η* ^2^_partial_s <0.02, BF_10_s < 0.51) and Time × Group interaction (*F*s <0.59, *p*s > .448, *η* ^2^_partial_s <0.01, BF_10_s < 0.36). These results indicated that both learning groups increased emotional arousal and valence for the second‐time listening.

### Physiological similarities of skin conductance and heart rate among participants

**FIGURE 4:**
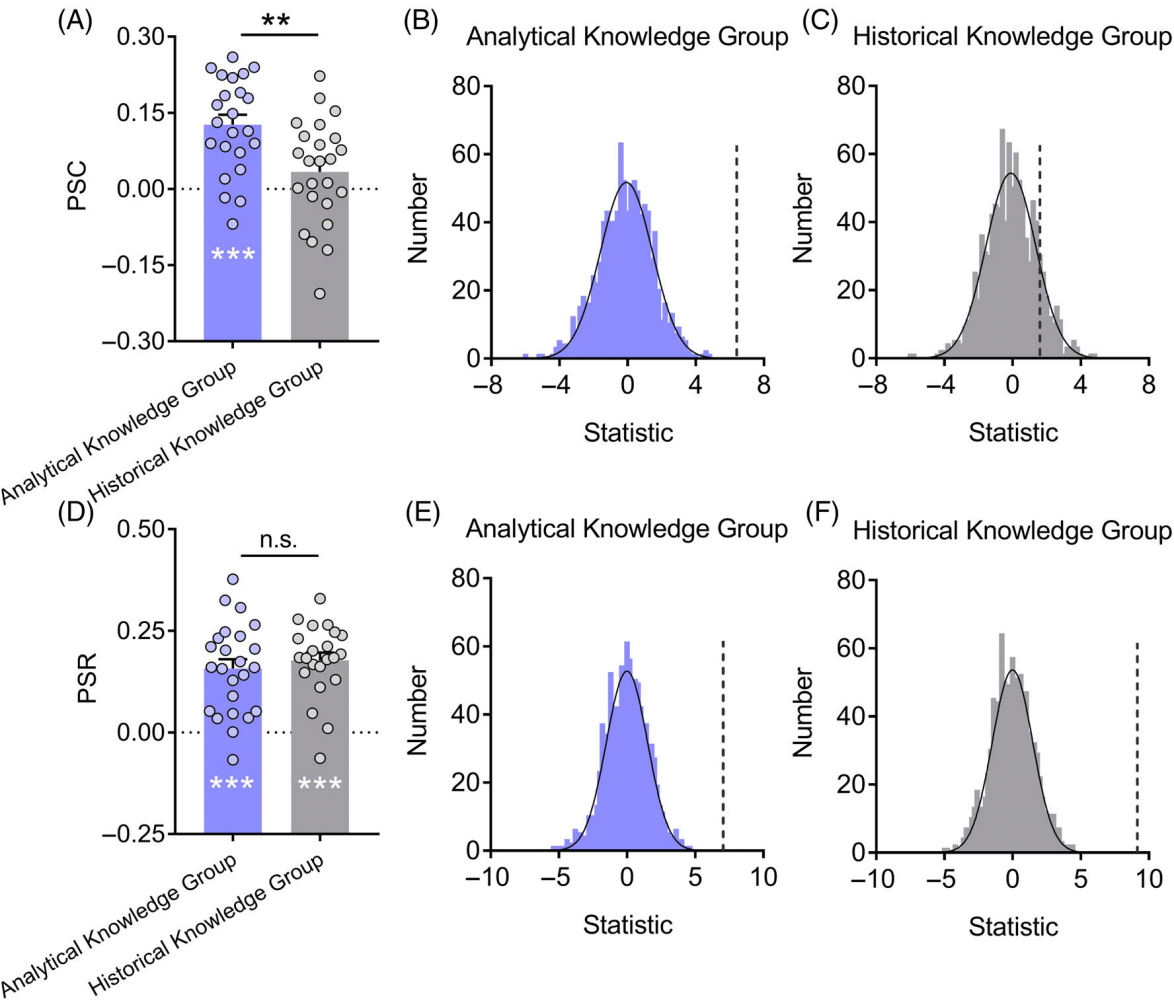
Physiological similarities of skin conductance (PSC) and heart rate (PSR) among participants. (A) Only the analytical knowledge group showed significant PSC. PSC were significantly higher for the analytical knowledge group (*n* = 23) than for the historical knowledge group (*n* = 24). Permutation tests validated the significant PSC (B) in the analytical knowledge group, (C) but not in the historical knowledge group. Histograms show the frequency distribution of null *t*‐statistics; black vertical lines denote the observed *t*‐statistic. (D) PSR did not differ between the analytical knowledge group (*n* = 24) and the historical knowledge group (*n* = 22). Permutation tests validated the significant PSR (E) in the analytical knowledge group and (F) in the historical knowledge group. Error bars represent standard errors of the mean, and “n.s.” indicates non‐significant (*p* > .05). **p < .01; ***p < .001.

We next explored how acquisition of musical knowledge influenced physiological responses. The participants in the analytical knowledge group showed significant physiological similarity of skin conductance (PSC) (0.13 ± 0.09, *t*(22) = 6.50, *p* < .001, Cohen's *d* = 1.35 [95% CI: 0.77, 1.91], BF_10_ = 1.19 × 10^4^; see Figure 4A). There was little evidence for the existence of PSC in the historical knowledge group (0.03 ± 0.10, *t*(23) = 1.57, *p* = .130, Cohen's *d* = 0.32 [95% CI: −0.09, 0.73], BF_10_ = 0.65). As expected, PSC was significantly higher in the analytical knowledge group than in the historical knowledge group (*t*(45) = 3.26, *p* = .002, Cohen's *d* = 0.95 [95% CI: 0.34, 1.55], BF_10_ = 16.51), indicating that the learning of analytical knowledge led to greater physiological alignment. To validate the significant PSC in the analytical knowledge group, we conducted a permutation test, which supported the findings (*p* = 2.62 × 10^−5^; see Figure 4B), but not in the historical knowledge group (*p* = .260; see Figure 4C).

The parallel analyses were conducted on the data of heart rate. Significant physiological similarity of heart rate (PSR) was observed in both groups (*t*s > 6.92, *p*s < .001, Cohen's *d*s > 1.41, BF_10_s > 3.83 × 10^4^; see Figure 4D). The degree of PSR was comparable between the two groups (0.16 ± 0.11 vs. 0.18 ± 0.09, *t*(44) = −0.66, *p* = .514, Cohen's *d* = 0.19 [95% CI: −0.77, 0.39], BF_10_ = 0.35). Additionally, permutation tests supported significant PSR in both groups (*p*s <9.89 × 10^−10^; see Figure 4E,F).

### The relation between physiological similarity and music liking change

**FIGURE 5:**
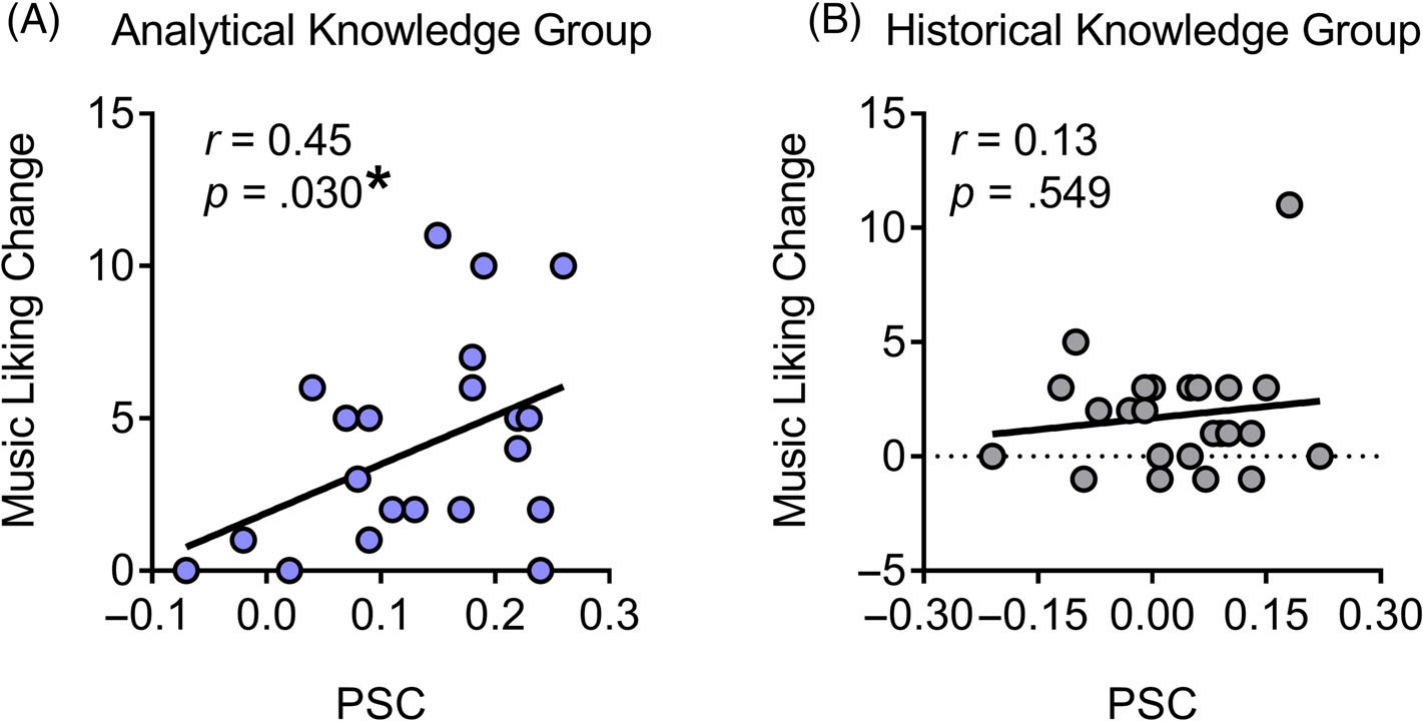
Correlation between physiological similarities of skin conductance (PSC) and music liking change. (A) Positive correlation in the analytical knowledge group. (B) No correlation in the historical knowledge group. Music liking change was indexed by the changed liking score (post‐liking minus pre‐liking). *p < .05.

We then investigated the relation of physiological similarity with the change in music liking. A series of Pearson correlation analyses found a significant correlation between PSC and music liking change in the analytical knowledge group (*r*(21) = 0.45, *p* = .030 [95% CI: 0.05, 0.73], BF_10_ = 2.41; see Figure 5A), but not in the historical knowledge group (*r*(22) = 0.13, *p* = .549 [95% CI: −0.29, 0.51], BF_10_ = 0.30; see Figure 5B). Moreover, multiple regression analysis revealed that PSC independently predicted music liking change, even considering the effect of knowledge acquisition type (*β* = 0.31, *R* ^2^ = 7.70%, *F*(1, 44) = 4.22, *p* = .046).

Similar analyses conducted on PSR showed no significant correlation in either the analytical or the historical knowledge group (*r*s < −0.004, *p*s > .608 [95% CI: −0.41, 0.40], BF_10_s < 0.30).

### The mediation effect of physiological similarity on the relation between musical knowledge and music liking change

The above results showed that knowledge acquisition groups differed in music liking change and physiological similarity. Such similarity correlated with music liking in the analytical knowledge group, but not in the historical knowledge group. These findings suggested there might be a mediating influence of physiological similarity in the association of knowledge learning groups with their music liking change. To test this, we performed a mediation analysis using PROCESS Model 4, with emotional empathy ability as a covariate to control for individual differences associated with PSC (analytical knowledge group: *r* = 0.02, *p* = .947 [95% CI: −0.40, 0.42], BF_10_ = 0.26; historical knowledge group: *r* = 0.59, *p* = .002 [95% CI: 0.25, 0.81], BF_10_ = 21.29).

**FIGURE 6:**
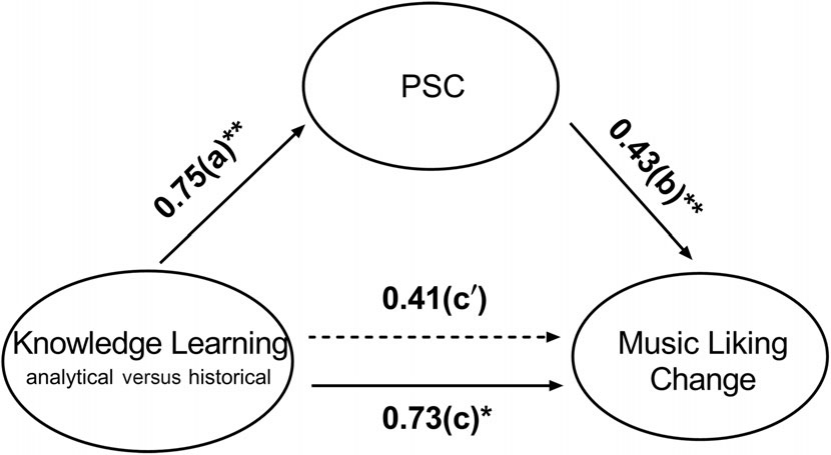
The mediation effect of physiological similarities of skin conductance (PSC). The effect of knowledge learning on music liking change was fully mediated by PSC, controlling for emotional empathy ability. All path coefficients are standardized. *p < .05; **p < .01.

The results confirmed our expectations (see Figure 6). Notably, (i) knowledge acquisition predicted music liking change (path *c* = 0.73, *p* = .013), but (ii) the relationship between knowledge acquisition and change in music liking weakened and became insignificant when including PSC as a mediator (path *c'* = 0.41, *p* = .170). The mediation effect was significantly different from zero (*β* = 0.32, 95% CI for *β* = [0.03, 0.62]). As a control variable, emotional empathy had a significant impact on PSC (*β* = 0.35, *p* = .009), but not on music liking change (*β* = −0.28, *p* = .059). In short, the physiological similarity of skin conductance fully mediated the effect of knowledge acquisition on music liking. Analytical knowledge brought listeners into greater physiological concordance, and it amplified the enjoyment of the music.

Similar analyses conducted on PSR showed no significant mediation effect (*β* = 0.008, 95% CI for *β* = [−0.06, 0.20]).

### The relation between physiological similarity and knowledge similarity

**FIGURE 7:**
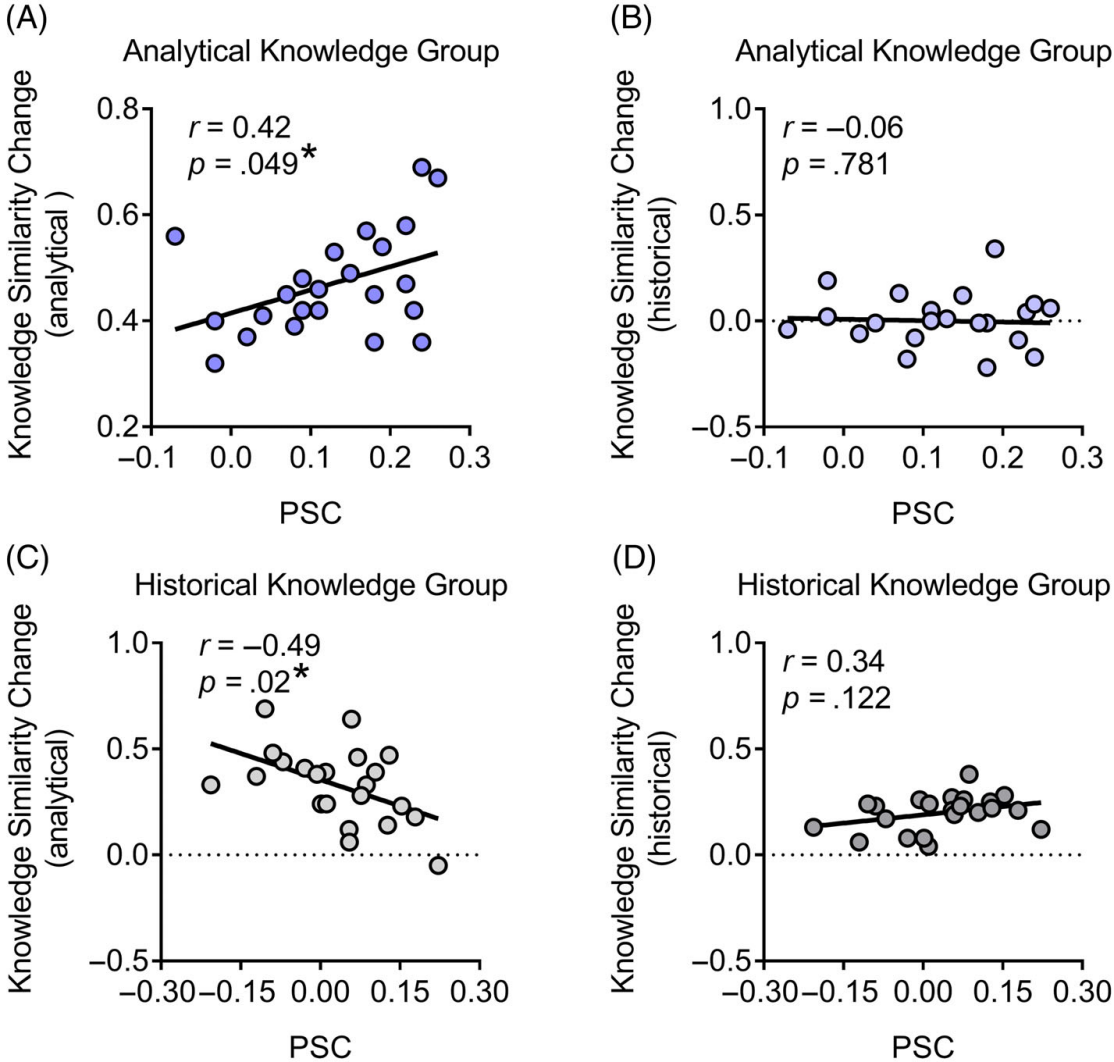
Correlation between physiological similarities of skin conductance (PSC) and knowledge similarity change. In the analytical knowledge group, PSC positively correlated with (A) change in analytical knowledge similarity, but not with (B) that in historical knowledge similarity. In the historical knowledge group, PSC negatively correlated with (C) change in analytical knowledge similarity, but not with (D) that in historical knowledge similarity. Knowledge similarity change was indexed by the changed value (post‐knowledge similarity minus pre‐knowledge similarity). Error bars represent standard errors of the mean. *p < .05.

Finally, we explored whether there was an association between physiological similarity and knowledge similarity among participants. In the analytical knowledge group, Pearson correlation analyses showed that PSC positively correlated with change in analytical knowledge similarity (*r*(21) = 0.42, *p* = .049 [95% CI: 0.00, 0.71], BF_10_ = 1.60; see Figure 7A), but not with change in historical knowledge similarity (*r*(21) = −0.06, *p* = .781 [95% CI: −0.46, 0.36], BF_10_ = 0.27; see Figure 7B). To compare the strength of these correlations, we used the algorithm implemented in the cocor R package (Diedenhofen & Musch, 2015). The results showed that the correlation between PSC and analytical knowledge similarity was significantly stronger than that between PSC and historical knowledge similarity (*z* = 2.05, *p* = .040). The findings indicated that physiological similarity was related to a shared analytical understanding of music.

In the historical knowledge group, our analyses showed that PSC negatively correlated with change in analytical knowledge similarity (*r*(22) = −0.49, *p* = .020 [95% CI: −0.09, −0.76], BF_10_ = 3.28; see Figure 7C). There was little evidence for the correlation of PSC with change in historical knowledge similarity (*r*(22) = 0.34, *p* = .122 [95% CI: −0.10, 0.67], BF_10_ = 0.81; see Figure 7D). These correlations differed significantly (*z* = 3.46, *p* = .001).

We also examined the relations between PSR and knowledge similarity. Little evidence was shown for this correlation in the analytical knowledge group (*r*s < −0.16, *p*s > .060 [95% CI: −0.54, 0.27], BF_10_ < 1.36). Although there was a significant correlation in the historical knowledge group (historical knowledge: *r*(22) = 0.44, *p* = .041 [95% CI: 0.02, 0.73], BF_10_ = 0.53), this was not different from the other correlation (analytical knowledge: *r*(22) = 0.32, *p* = .146 [95% CI: −0.12, 0.65], BF_10_ = 0.71; *z* = 0.45, *p* = .651).

## DISCUSSION

In this study, our key question is how musical knowledge influences music enjoyment. We did this by comparing the effect of acquiring two types of musical knowledge—analytical information about the music itself versus historical information about the music's background—on music liking. We measured physiological responses to see if analytical knowledge made listeners more aligned, thus making them like the music more. We found that learning analytical information, relative to historical information, could increase music liking. When learning analytical information, participants had similar physiological responses, both in skin conductance and heart rate. The physiological similarity of skin conductance, correlated with analytical knowledge similarity, fully mediated the effect of knowledge acquisition on music liking.

First, musical knowledge causes music liking. It might be that other factors in the two knowledge groups influence music liking. This study showed both groups felt comparable emotions, engagement, and teacher liking after learning. We did find the historical knowledge group reported that learning was more difficult. However, in both groups, learning difficulty did not significantly correlate with knowledge acquisition, knowledge similarity, and physiological similarity (*r*s <0.36, *p*s > .097), or music liking (*r*s <0.04, *p*s > .160). Similarly, engagement in learning did not significantly correlate with knowledge acquisition (*r*s <0.20, *p*s > .270), physiological similarity (*r*s <0.27, *p*s > .218), or music liking (*r*s <0.17, *p*s > .421). Thus, we argue that the group difference in music liking results from acquiring different musical knowledge.

Consistent with our findings, some studies in other arts, such as drawing, also showed the relationships between knowledge acquisition and aesthetic emotion. For instance, understanding art construction rules and cultural background could enhance appreciation (Gordon & Holyoak, 1983; Temme, 1992). If artworks had elaborate titles, there would be increased enjoyment and interest (Millis, 2001; Russell, 2003). Music was evaluated more favorably in the presence of titles than in their absence (Anglada‐Tort et al., 2019). Thus, expertise in art can produce more rewarding experiences (Leder et al., 2004). A review study proposed that art appreciation involves a dynamic interaction between bottom‐up perceptual and top‐down cognitive processes (Kirsch et al., 2016). All these studies suggest that art appreciation is not only influenced by the artwork itself, but also by the cognition of the appreciator.

Second, acquiring analytical knowledge elicited greater physiological similarity, as indicated by concordant changes in skin conductance. The physiological similarity mediated the effect of knowledge learning on music liking. In other studies, neurophysiological synchronization has also been shown to correlate with music liking. For instance, listeners exhibited greater neurophysiological synchrony when listening to preferred music excerpts (Chabin et al., 2022; Czepiel et al., 2021; Tschacher et al., 2023). Connections between listeners' brains are also related to the popularity of musical performances (Leeuwis et al., 2021). Furthermore, inter‐brain coherence between the audience and performer forecasted music popularity (Hou et al., 2020). We build on these findings by further showing that acquiring analytical knowledge can align physiological reactions and increase music liking. The physiological similarity has been found to be associated with emotional ability as well as social cohesion. For instance, when infants' physiological responses matched those of caregivers, their emotion regulation improved and negative reactivity decreased (Abney et al., 2021; Pratt et al., 2015). Interpersonal synchrony fosters social benefits (Hoehl et al., 2021), such as rapport (Miles et al., 2009), cooperation (Behrens et al., 2020; Prochazkova et al., 2018; von Zimmermann & Richardson, 2016), and affiliation (Hove & Risen, 2009; Lumsden et al., 2014). Thus, when people share similar understanding, their physiological responses become more coordinated, strengthening mutual rewards such as the enjoyment of music.

Our study did not find a significant correlation for the physiological similarity of heart rate. Skin conductance and heart rate are the most common physiological responses. Skin conductance reflects only the sympathetic nervous system response, but heart rate reflects both the sympathetic and parasympathetic systems (Eisenbarth et al., 2016). Skin conductance is consistently related to emotional arousal, but the relationship between heart rate and arousal is more complex. Specifically, it has been reported that both heart rate accelerations and decelerations are related to arousal (Vaughan & Lanzetta, 1981). This could be because arousal is linked to the body being ready for action (the defense reflex) and concentration (the orienting reflex). They are related to heart rate accelerations and decelerations, respectively. A more complex relationship with arousal may reduce some participants' physiological similarity of heart rate (Stuldreher et al., 2020). Our additional finding showed that emotional arousal positively correlated with the physiological similarity of skin conductance rather than heart rate (see details in Supplementary Materials).

Third, there was a positive correlation between physiological similarity and similarity in analytical knowledge. The more knowledge listeners shared about the music, the more similar the physiological responses were. This study suggests that listeners became emotionally aligned when acquiring analytical knowledge. Since this knowledge reflected emotional expressions of music, listeners likely felt emotionally attuned during learning. The Neurocognitive Model of Emotional Contagion (NMEC) theory proposes that physiological responses enable shared emotional representations and affective alignment (de Waal & Preston, 2017; Prochazkova & Kret, 2017). People who watched movies together also had similar physiological responses and emotions (Golland et al., 2015). Future research may examine different emotional aspects of physiological concordance while acquiring musical knowledge.

In addition to our primary focus on physiological similarity, we also examined conventional individual‐level physiological measures such as skin conductance and heart rate. However, these measures showed no significant differences between groups, nor the associations with music liking (see details in Supplementary Materials). This lack of significant findings at the individual level suggests that such measures may not fully capture the subtleties of how musical knowledge influences music appreciation. Therefore, our analysis pivoted to inter‐subject comparisons, which proved to be more sensitive in examining the impact of knowledge acquisition on music enjoyment. This approach aligns with the SAME model, which conceptualizes music appreciation as a fundamentally shared experience.

The SAME model, described in Section 1, can be used to explain how analytical knowledge increases music liking. People like the music they know well (Peretz et al., 1998; Soley & Hannon, 2010) and recognize its emotions better (Elfenbein & Ambady, 2002, 2003; Laukka et al., 2013, 2016). The SAME model suggests that music is rewarding when we catch the conveyed emotional meaning. This study adds to this model by explaining that shared analytical knowledge of how music expresses emotions helps us feel emotional synchronization with others. We can sense emotions more strongly when learning more about music itself. We understand how musical elements create emotion and meaning. By gaining this knowledge, we catch the emotions in a piece of music. This emotional understanding makes us like the music more because it feels good to share feelings with others. In short, acquiring knowledge about emotional expressions with musical elements helps us align with others, which leads to more enjoyment.

The current findings validate and extend the SAME model by showing that acquiring analytical knowledge increased music liking, which correlates with more shared physiological responses. Analytical knowledge likely enabled learners to better grasp the emotional expressions in the music. This led to more aligned physiological reactions, indicating shared embodied experiences. The correlation between physiological and knowledge similarity further suggests that analytical knowledge cultivates mutual understanding of the conveyed emotions in the music. This makes the appreciation process more rewarding by eliciting greater alignment in embodied experiences. In all, analytical knowledge teaching amplifies shared affective motion experience, lending support to the SAME model hypothesis regarding the mechanism of music appreciation.

Our finding showing enhanced music liking after analytical knowledge acquisition also aligns with and extends previous evidence on the influential role of source information in aesthetic evaluation (e.g., Anglada‐Tort & Müllensiefen, 2017; Anglada‐Tort et al., 2019). Importantly, we reveal that analytical knowledge targeting core aspects of the music has a greater appreciative effect than historical knowledge, highlighting the significance of this type of knowledge distinction. This finding provides novel insight into the mechanism underlying music appreciation. It also informs practical strategies for music pedagogy—while students learn both analytical and historical knowledge in classes, placing greater emphasis on analytical facets that promote essential understanding of the music could be more effective for enhancing music enjoyment.

As discussed above, understanding the music itself is imperative for appreciation. Our results revealed a weaker effect of historical knowledge on music liking compared to analytical knowledge. Nevertheless, certain contextual knowledge like historical background may also contribute to the appreciation for some listeners. Future research could further examine individual differences in how historical knowledge influences music enjoyment. For instance, some listeners may not be interested in the historical background of musical compositions. If the knowledge fails to connect with the listener's personal experiences, its impact on music appreciation may be small. A systematic investigation of the circumstances where historical knowledge has more significant appreciative effects deserves further study.

Our findings that analytical knowledge significantly enhances music appreciation have important practical implications. Firstly, these findings provide direct guidance for music education. Incorporating more analytical knowledge about music elements and emotional expressions into the curriculum can enhance students' understanding and appreciation of music. Educators can achieve this through curriculum design that focuses on the internal structure and emotional conveyance of music, rather than just its historical background. Additionally, utilizing multimedia teaching tools, such as videos and interactive software, can help students grasp the elements and emotional expressions of music more intuitively. Secondly, the application of analytical knowledge in music therapy holds potential. Music therapists can use these findings to teach patients analytical knowledge, helping them to better perceive and understand music, thereby achieving therapeutic effects. Integrating the analysis of musical elements into therapy sessions can assist patients in expressing and releasing emotions through music. Tailoring the teaching of analytical knowledge to patients' personal interests and needs can further enhance the effectiveness of therapy. Future research could explore the effects of analytical knowledge teaching/learning across different music genres, examine responses among various genders and age groups, and evaluate the practical implementation of these strategies in educational and therapeutic settings.

There are limitations to this study. Firstly, this study concerns mainly the effects of analytical versus historical knowledge acquisition on music appreciation, using one music piece as an example. We acknowledge that music in different styles can elicit more complex physiological reaction patterns, interacting with individual preferences and exposure experiences. Future research should implement various musical pieces and styles to validate and generalize our conclusion on the relationship between knowledge type and music liking. Meanwhile, using approaches to expose participants to different conditions, we need to be careful of “order” and “carry‐over” factors that bias the findings (Maclure, 1991; Mills et al., 2009). Secondly, this study included only female participants. Females react more strongly to music. Females, compared with males, have different music preferences (Christenson & Peterson, 1988; Colley, 2008; Kamenetsky et al., 1997; Schwartz & Fouts, 2003) and emotional responses (Coffman et al., 1995; Kamenetsky et al., 1997; Panksepp, 1995). We do not know how knowledge influences males' music enjoyment, which needs more study in later research.

Lastly, we acknowledge that without a control group, it is impossible to completely rule out the influence of the mere exposure effect on the results. The mere exposure effect suggests that people's preferences and positive attitudes toward stimuli increase after repeated exposures. Hence in the current study, the increased music liking in the second‐time listening could be attributed to both learning effects and mere exposure effects. However, there is evidence that the learning effect outweighs the mere exposure effect in explaining the results. First, there was a significant difference in music liking increase between the two learning groups in the post‐learning listening, which is less likely to be explained by simple repetition. Second, the mediating effect of physiological similarity also supports the impact of learning on the results. In future studies, multiple condition groups could be designed (e.g., repetition, historical, analytical groups) to effectively examine the influences of learning versus exposure effects on music appreciation. This would also improve the current research.

In summary, our study shows that acquiring analytical knowledge, as opposed to historical knowledge, leads to a greater increase in music liking. Similar skin conductance, relating to understanding the music similarly, can mediate the effect of knowledge acquisition on music liking. Our findings suggest a psychophysiological mechanism explaining musical knowledge's impact on music enjoyment. This study gives new insight into how knowledge influences listening experiences at cognitive and physiological levels. It has important implications for research in learning, emotional psychology, and musical education. Future research can explore how various types of knowledge affect music appreciation by combining factors from music properties, learners' characteristics, instructional methods, or cultural contexts.

## CONFLICT OF INTEREST STATEMENT

The author(s) declared that there were no conflicts of interest with respect to the authorship or the publication of this article.

## ETHICS STATEMENT

Written informed consent was obtained after the experimenter explained the study. This study was approved by the University Committee of Human Research Protection (HR 147‐2019) at East China Normal University.

## Supporting information


**Data S1.** Supplementary Information.
