# Supplementary material for: Acquiring musical knowledge increases music liking: Evidence from a neurophysiological study
Source: Psych J. 2024 Jul 21;13(6):927–42. doi: 10.1002/pchj.791 (PMC11608780; doi:10.1002/pchj.791)
Supplement: Supplementary file 1 — Data S1. Supplementary Information. [file PCHJ-13-927-s001.doc]

**Learning Musical Knowledge Increases Music Liking:**

**Evidence from a Neurophysiological Study**

Yingying Hou^1^, Bei Song^2^, Yi Zhu^1^, Linwei Yu^1^ & Yi Hu^1*^

^1^ Shanghai Key Laboratory of Mental Health and Psychological Crisis Intervention, School of Psychology and Cognitive Science, East China Normal University, Shanghai 200062, China

^2^ Department of Art, Harbin Conservatory of Music, Harbin 150000, China

**^*^ Corresponding Author**

Yi Hu, Shanghai Key Laboratory of Mental Health and Psychological Crisis Intervention, School of Psychology and Cognitive Science, East China Normal University, 3663 North Zhongshan Road, Shanghai 200062, China.

Phone number: 13916582896; E-mail: [yhu@psy.ecnu.edu.cn](mailto:yhu@psy.ecnu.edu.cn)

# Supplemental Materials

# Supplemental Methods

### Pre-recording Session for the Violinist and the Teacher

During the pre-recording session, the violinist and the teacher arrived at the laboratory on separate days. The violinist performed a selected music piece, while the teacher taught musical knowledge (analytical vs. historical). The violinist's performance and the teacher's lessons were recorded on video.

The selected music piece had not been previously performed by the violinist in formal settings, such as concerts. Thus, he was given ample time to practice at home until he was satisfied with his performance. Additionally, he was required to familiarize himself with analytical and historical knowledge in the instructional materials provided by the teacher. It might help the violinist fully immerse himself in the music piece during his performance. Upon arrival at the laboratory, the violinist underwent two knowledge tests, one for analytical and one for historical knowledge, similar to the tests administered to the learners before and after learning (see section Experimental Protocol). The violinist received full marks on both tests, indicating the best acquisition of musical knowledge. Subsequently, the violinist was positioned in front of a video camera, instructed to maintain a neutral facial expression while performing the music, and given three minutes to prepare. Two experimenters observed his performance, which lasted for 190 seconds.

Upon arrival, the teacher was guided to a quiet room with a laptop computer on the table. The papers handed out by the teacher were placed beside the computer. The teacher was instructed to sit upright at an appropriate distance of 60 centimeters from the computer. She was advised to conduct the lesson smoothly without too many facial expressions or movements. The initial preparation time for the teacher was approximately 5 minutes. Then, she delivered two lessons in front of the computer. Lesson 1 (689 s) and Lesson 2 (627 s) was with analytical and historical knowledge respectively. A 3-minute break was provided between the two lessons. A freely available screen recording software (Apowersoft 2) was used to record the lessons via a split-screen video, in which the courseware was displayed on the main screen and the teacher's upper body was located in the upper right corner.

**Details about Two Types of Musical Knowledge Teaching**

*Analytical knowledge teaching*

The analytical knowledge teaching consists of four sections: introduction, main content, summary, and ending. The introduction briefly describes the mood and name of the music piece "Dreaming" to attract learners' attention.

The main content focuses on analytical aspects of the music itself, including:

1. Emotional theme: yearning for a happy childhood
2. Instrument timbre: violin, which has a tender timbre suitable for conveying delicate emotions
3. Tempo: very smooth and slackened to give listeners a feeling of time slowly passing by
4. Dynamics: soft at the beginning, getting louder, then softer again, forming waves of sound
5. Form structure: three-part form with symmetric structure between main theme (A) and recurrence (A') to achieve a sense of beauty

The teacher explains the above analytical knowledge with examples of music excerpts inserted. For instance, when introducing the three-part form, the excerpt of each section is played respectively.

In the summary and ending, the teacher highlights the key points and profoundly reflects on the meaning of life contained in this short piece.

The text transcript for the analytical teaching content:

[The teacher’s words] “Through our recent listening experience, you must have felt that the mood of this music piece is very tranquil, sweet, soft, and affectionate. The name of this piece, just like the musical atmosphere it creates, is called 'Träumerei.' Next, let us delve into this composition from five aspects: the theme, the instrument used, the tempo and dynamics, and the structure of the music.

The composer of 'Träumerei' is Robert Schumann, a renowned composer from the German Romantic period. Secondly, regarding the theme of 'Träumerei,' this musical piece was composed by Schumann in 1838 as part of his piano suite 'Scenes from Childhood.' Although titled 'Scenes from Childhood,' these compositions were not specifically written for children but to represent adults' reminiscences of childhood. They express the composer's nostalgia and fondness for the blissful times of childhood. Thus, the entire piece, like its title 'Träumerei,' seems to create a warm, sweet, and slightly melancholic dreamworld for us.

To better convey these thoughts and feelings, we chose the violin for this piece. The violin, a bowed string instrument, produces sound by the bow rubbing against the strings. Its tone is very delicate and beautiful, with a strong lyrical quality, vividly depicting the composer's emotional state and creating a warm, serene, and deeply moving musical atmosphere, subtly drawing the listener into a light and ethereal dream world.

Regarding the tempo of 'Träumerei,' you must have noticed the overall slowness of the piece. This slow tempo is used to create a musical sensation as if we are sitting by the riverbank of time, watching time flow gently by. The happy times of childhood slowly pass by and recede in this process.

About the dynamics of the music, let's first understand what musical dynamics are. Dynamics refer to the strength of force applied to the bow when playing the violin, determining the volume of the music. That is, the stronger the force applied to the bow, the louder the music, and vice versa. If you listen carefully, you will find that the dynamics of the entire piece actually vary in intensity. Initially, the music starts softly, gradually becomes louder, then diminishes in the middle, increases again, and gradually fades until the end. In this way, the whole piece, like ripples of water, undulates and creates a sense of ups and downs.

The final aspect is the structure of this musical piece, which, you might not know, has a compositional structure just like the literary works we read, even including plot development. 'Träumerei' is structured in a very common three-part form in musical compositions, meaning the entire piece is divided into three sections. Let's listen to the first section. (Music is playing.) Now, let's listen to the second section. (Music is playing.) You can feel that the melody and rhythm of the second section are somewhat different from the first, showing some changes. Then, let's listen to the third section. (Music is playing.) It's easy to find that the third section is essentially the same as the first. That is to say, the third section is actually a repetition and reprise of the first.

So, why did the composer use such a structure for this music piece? The reason for adopting this compositional method is to create a contrast between the first and second sections, and a symmetry between the first and third sections. This way, the listeners neither feel bored due to excessive repetition nor overwhelmed by too many changes, leaving a deep impression in their minds. Isn't this a clever design? In fact, symmetrically structured things are ubiquitous in our lives, such as interior layouts, architectural designs, and even our human faces and body structures. Our lives are filled with symmetrical elements, and the musical structure of 'Träumerei' is a concentrated expression of this beauty of symmetry.

In summary, although 'Träumerei' is brief in length, it encapsulates profound contemplations about life and exudes a sense of melancholy due to the passing of childhood. Listening to this piece is like watching a boat carrying our happy childhood memories slowly drift away. Despite the reluctance to let go, all we can do is silently wave goodbye...”

*Historical knowledge teaching*

The historical knowledge teaching also consists of four sections: introduction, main content, summary, and ending. The introduction describes the music piece to be appreciated and positions it in the artistic movement of musical romanticism.

The main content focuses on relevant historical knowledge about Romanticist music, including:

1. Definition and key ideas of musical Romanticism, which values individuality and free emotional expression
2. Timeframe (early 19th century) and main artistic fields it influenced
3. Three representative Romanticist composers and their masterpieces - Beethoven's Fate Symphony symbolizing his struggle against destiny; Chopin's Nocturnes expressing delicate feelings; Tchaikovsky’s Swan Lake ballet music depicting a romantic fairy tale.

In the summary and ending, the teacher concludes that Romanticist music left many brilliant musical works.

The text transcript for the historical teaching content:

[The teacher’s words] “The music piece we just appreciated is the 'Träumerei' composed by Robert Schumann, a prominent figure of the Romantic era in music composition. This piece is a product of the Romantic period in the history of music development.

Romanticism is an artistic creation method that uses rich imagination and exaggerated expression to reflect real life and emotions. It emerged during the French Revolution, a period advocating the ideals of 'liberty, equality, and fraternity,' which directly influenced people's demand for personal liberation and emotional expression, becoming the core ideology of Romanticism. Consequently, artists of this period greatly revered subjective feelings, love for nature, and aspiration for the future. Originating in the early 19th century, Romanticism profoundly influenced various fields, including music, art, dance, drama, literature, and architecture, spanning approximately from 1800 to 1900.

Music of the Romantic era placed significant emphasis on the expression of rich, personal emotions and thoughts. Therefore, composers of this era infused their personal feelings and thoughts into their music, making their works more free, unrestrained, and imaginative, with melodies that were broad and extended. Their music was richly diverse in content and highly individualized. Many renowned musicians represented this period, with Ludwig van Beethoven being one of the most familiar. Beethoven, one of the greatest musicians from Germany, was revered as the 'Master of Music.'

Under his father's strict training, Beethoven began learning music at a very young age and soon demonstrated extraordinary talent. For instance, he could play the piano at the age of four and was recognized as a musical prodigy. He began performing on stage at the age of eight and published his first piano composition at eleven. One of his most famous works is the Symphony No. 5 in C minor, commonly known as the 'Fate Symphony,' which is highly regarded and frequently performed worldwide. Beethoven started conceptualizing and writing the 'Fate Symphony' in 1804. At that time, he was nearly deaf due to illness, a devastating blow for any musician. Additionally, the love of his life left him due to social class differences. These successive emotional traumas brought Beethoven to the brink of death. However, he did not succumb to despair; his love for life and relentless pursuit of art helped him overcome his personal suffering and despair. In fact, his hardships became the source of inspiration for his compositions. The 'Fate Symphony' embodies Beethoven's lifelong struggle against fate.

Another famous musician of the Romantic era was the Polish composer Chopin. Chopin began composing music at the age of seven and started performing on stage at eight. From the age of twelve, he studied composition and music theory at the Warsaw National Higher School of Music. By the age of nineteen, Chopin embarked on a European tour as a composer and pianist. Later, after the failure of the Warsaw Uprising, he settled in Paris, mainly engaging in music teaching and composition. Unfortunately, he passed away in Paris at the age of 39 due to tuberculosis.

During his short life, Chopin composed about two hundred music works, with his Nocturnes being the most representative. The Nocturne is a piano music genre mainly used to portray the tranquility of the night and people's delicate inner emotions, typically played at a very slow tempo with beautiful and lyrical melodies. Chopin's personality, marked by elegance and nobility, naturally expressed deep emotions in the sweet melodies of the Nocturnes. Due to his physical frailty and illness, he was not suited for performing loud, grand narrative music but was more adept at expressing the delicate emotions and charming temperament of the Nocturnes. Therefore, he composed 21 exquisite Nocturnes throughout his life, with their stylistic changes reflecting the development of his life course, culminating in a beautiful final chapter of his life. As most of his music works are poetic and picturesque, Chopin is acclaimed as the 'Romantic Piano Poet.'

Another great musician of the Romantic era was the Russian composer Pyotr Ilyich Tchaikovsky, known as the 'Master of Russian Music.' His first ballet, 'Swan Lake,' with a musical theme inspired by folk tales, narrates the story of a prince who nearly falls for the deception of a black swan but eventually discovers and defeats the evil sorcerer, restoring the white swan to her original form as a princess, leading to a happy union. The accompanying music of the ballet 'Swan Lake,' like romantic lyric poems, perfectly portrays the characters' personalities and inner emotions, significantly enhancing and propelling the ballet's storyline. In summary, Romanticism had a substantial impact on all forms of art, including music, dance, and drama. The musical works mentioned above are classic masterpieces left by some of the most brilliant musicians in human history..”

### Questionnaire Items about Empathy for the Music

(5 items, from 0 = not at all to 10 = very intensely)

1. You are completely absorbed in this musical composition.

2. You are deeply touched by the music.

3. You experience the intended emotions conveyed by the performer's rendition.

4. You establish an emotional connection with the performer.

5. You envision yourself as the artist, performing the music as if it were your own.

**Supplemental Results**

**Individual-Level Physiological Measures**

In our study, alongside the primary focus on physiological similarity, we conducted an in-depth analysis of individual-level physiological measures, including skin conductance and heart rate, to capture the comprehensive physiological effects induced by the learning process.

Similar to our approach with physiological similarity, we extracted and preprocessed physiological data during the knowledge learning phase. For each participant, we averaged the data for skin conductance level and heart rate separately. Conducting two-sample *t*-tests to assess differences between the analytical and historical knowledge groups, we found no significant differences in the averaged skin conductance level and heart rate (*t*s < 0.92, *p*s > .363). Additionally, our analysis revealed no significant associations between these physiological measures and music liking in either group (analytical: *r*s < 0.16, *p*s > .469; historical: *r*s < 0.06, *p*s > .791).

The absence of significant findings suggests that traditional measures like individual-level skin conductance and heart rate, while commonly used as indicators of emotional and arousal states, may not have the requisite sensitivity to discern the subtle emotional shifts induced by different types of musical knowledge. This realization led us to shift our emphasis to inter-subject physiological similarity. This focus more aptly aligns with the concept of shared experiences highlighted in the Shared Affective Motion Experience (SAME) model, offering a more suitable lens through which to examine the group-level impact of musical knowledge on appreciation.

**Analysis of the Relations of Emotional Arousal with Physiological Similarity**

We investigated the correlation between emotional arousal and physiological similarity. We assessed participants' emotional arousal using an 11-point Likert scale (see Experimental Protocol for details). It should be noted that there was one missing data in both the analytical and historical knowledge learning groups.

Pearson correlation analyses revealed that emotional arousal positively correlated with physiological similarity of skin conductance (PSC) (*r* (44) = 0.33, *p* = .025) but not with that of heart rate (PSR) (*r* (45) = -0.23, *p* = .131). These two correlations were significantly different from each other (*z* = 2.75, *p* = .006). After controlling for Group, partial correlation analyses showed a marginally significant positive correlation between emotional arousal and PSC (partial *r* (40) = 0.27, *p* = .083), and a non-significant correlation for PSR (partial *r* (40) = -0.22, *p* = .169). The former correlation was significantly greater than the latter (*z* = 2.24, *p* = .025). These findings suggest that the similar physiological reaction of skin conductance, rather than heart rate, is related to emotional arousal.
